# Platelet-derived growth factor-induced Akt phosphorylation requires mTOR/Rictor and phospholipase C-γ1, whereas S6 phosphorylation depends on mTOR/Raptor and phospholipase D

**DOI:** 10.1186/1478-811X-11-3

**Published:** 2013-01-11

**Authors:** Masoud Razmara, Carl-Henrik Heldin, Johan Lennartsson

**Affiliations:** 1 Ludwig Institute for Cancer Research, Science for life laboratory, Box 595, Biomedical Center, SE-751 24, Uppsala, Sweden

**Keywords:** PDGF, PI3K, mTOR, Rictor, Raptor, Akt, PLC, PKC, PLD, S6

## Abstract

Mammalian target of rapamycin (mTOR) can be found in two multi-protein complexes, i.e. mTORC1 (containing Raptor) and mTORC2 (containing Rictor). Here, we investigated the mechanisms by which mTORC1 and mTORC2 are activated and their downstream targets in response to platelet-derived growth factor (PDGF)-BB treatment. Inhibition of phosphatidylinositol 3-kinase (PI3K) inhibited PDGF-BB activation of both mTORC1 and mTORC2. We found that in Rictor-null mouse embryonic fibroblasts, or after prolonged rapamycin treatment of NIH3T3 cells, PDGF-BB was not able to promote phosphorylation of Ser473 in the serine/threonine kinase Akt, whereas Thr308 phosphorylation was less affected, suggesting that Ser473 in Akt is phosphorylated in an mTORC2-dependent manner. This reduction in Akt phosphorylation did not influence the phosphorylation of the S6 protein, a well established protein downstream of mTORC1. Consistently, triciribine, an inhibitor of the Akt pathway, suppressed PDGF-BB-induced Akt phosphorylation without having any effect on S6 phosphorylation. Thus, mTORC2 does not appear to be upstream of mTORC1. We could also demonstrate that in Rictor-null cells the phosphorylation of phospholipase Cγ1 (PLCγ1) and protein kinase C (PKC) was impaired, and the PKCα protein levels strongly reduced. Furthermore, interfering with the PLCγ/Ca^2+^/PKC pathway inhibited PDGF-BB-induced Akt phosphorylation. In addition, PDGF-BB-induced activation of mTORC1, as measured by phosphorylation of the downstream S6 protein, was dependent on phospholipase D (PLD). It has been shown that Erk1/2 MAP-kinase directly phosphorylates and activates mTORC1; in partial agreement with this finding, we found that a Mek1/2 inhibitor delayed S6 phosphorylation in response to PDGF-BB, but it did not block it. Thus, whereas both mTORC1 and mTORC2 are activated in a PI3K-dependent manner, different additional signaling pathways are needed. mTORC1 is activated in a PLD-dependent manner and promotes phosphorylation of the S6 protein, whereas mTORC2, in concert with PLCγ signaling, promotes Akt phosphorylation.

## Background

Platelet-derived growth factor (PDGF) stimulates proliferation, migration and survival of mesenchymal cells and plays a pivotal role during embryonic development and wound healing [1]. The biologically active form of PDGF consists of disulphide-linked dimers, PDGF-AA, -AB, -BB, -CC and –DD, which bind to two structurally similar tyrosine kinase receptors, i.e. PDGFRα and PDGFRβ [2,3]. PDGFRα binds all PDGF chains except PDGF-D, whereas PDGFRβ interacts only with PDGF B- and D-chains. The binding of the bivalent ligand induces dimerization and activation of PDGFRs, leading to autophosphorylation of tyrosine residues in the intracellular region [2]. Thereby, several signal transduction pathways are initiated, including phosphatidylinositol 3-kinase (PI3K), the Src tyrosine kinase, phospholipase Cγ (PLC), and several mitogen-activated protein (MAP) kinase cascades.

mTOR is the mammalian ortholog of the yeast serine/threonine kinase TOR which is involved in the regulation of various cellular functions, such as initiation of translation, cell growth and proliferation, ribosome biogenesis, transcription and cytoskeletal reorganization [4]. Dysregulation of mTOR signaling is frequently seen in cancer and has attracted attention as a therapeutic target [5,6]. mTOR is functional in two distinct complexes, namely mTORC1 and mTORC2 [7]. mTORC1 activity is controlled by the G-protein Rheb; Rheb-GTP promotes mTORC1 activity and the tuberous sclerosis complex 1/2 (TSC1/2) acts as a GTPase activating protein for Rheb, consequently inhibiting mTORC1 activity [8]. Generally, mTORC1 is described as being activated by growth factors through Akt-mediated phosphorylation which inactivates the TSC1/2 complex [8-10]. In addition, the TSC1/2 complex can also be phosphorylated and inhibited by AMPK, thus allowing the cellular energy status to impact mTORC1 activity [11]. mTORC1 is a rapamycin-sensitive complex, and includes the proteins Raptor (regulatory-associated protein of mTOR), mLST8, PRAS40 and Deptor [12]. Raptor acts as a scaffold and thereby controls mTORC1 activity. Established functions for mTORC1 are to phosphorylate 4EBP1 and activate S6-kinase, which in turn phosphorylates the S6 protein [13]. Phosphorylated S6 and 4EBP1 enhance protein translation. In mTORC2, mTOR occurs in a complex with Rictor (rapamycin-insensitive companion of mTOR), mLST8, mSin1, protor, Deptor and Hsp70 [14-17]. mTORC2 is primarily activated by growth factors, but the mechanism is largely unknown. It has recently been suggested that mTORC2 activation is dependent on PI3-kinase, but independent of Akt [18]. mTORC2 is able to phosphorylate Akt on Ser473, at least in some cell types [19]. Other substrates for mTORC2 include PKCα and paxillin [20]. mTOR can be activated by growth factor signaling, such as by PDGF, but the roles of mTORC1 and mTORC2 in PDGF-BB-induced signal transduction have not been established.

The serine/threonine kinase Akt is activated by PDGF-BB stimulation in a PI3-kinase-dependent manner. Activation of PI3-kinase generates PIP_3_ that can interact with and thereby translocate Akt to the plasma membrane, where it is activated by phosphorylation on Ser473 in a hydrophobic motif and Thr308 in the activation loop of the kinase domain [19,21,22]. Thr308 is phosphorylated by phosphoinositide-dependent protein kinase 1 (PDK1), whereas several candidates, including mTORC2, may perform the Ser473 phosphorylation [19,23-25]. Furthermore, the kinase responsible for the Ser473 phosphorylation may be different for different cell and receptor types. When activated, Akt transduces important survival signals that interfere with the apoptotic process, for example by inhibition of Foxo, Bad and caspase 9 [26-28].

Phoshoplipase Cγ catalyzes the hydrolysis of PIP_2,_ thus releasing the polar head group inositol-1,4,5-trisphosphate (IP_3_), while diacylglycerol (DAG) remains embedded in the plasma membrane [29]. IP_3_ release results in mobilization of Ca^2+^ from intracellular stores. Both DAG and Ca^2+^ participate in the activation of protein kinase C (PKC) family members, some of which require both DAG and Ca^2+^ (PKCα, β, γ), whereas others require only DAG (PKCδ, ε, η, θ) [30]. In addition, there are atypical PKC isoforms (PKCζ, ι) that are regulated by other means [31]. PLCγ is activated by direct SH2-domain-dependent interaction with activated tyrosine kinase receptors and subsequent phosphorylation [32,33]. Another phospholipase that is activated by receptor tyrosine kinases is phospholipase D (PLD). PLD acts by hydrolyzing phosphatidylcholine generating choline and phosphatidic acid [34] which is required for mTORC1 activation by mitogenic factors [35]. Regulation of PLD activity is complex and has been shown to involve small G-proteins, phosphatidylinositol 4,5-bisphosphate (PIP_2_), Ca^2+^ and kinases [36]. PDGF has been demonstrated to promote PLD tyrosine phosphorylation and activation by a mechanism involving the production of reactive oxygen species [37].

In this study, we have explored the role of mTOR in the regulation of PDGF-BB signaling. We found that Rictor, and hence mTORC2, promotes the PDGF-BB-induced phosphorylation of Akt at Ser473, as well as the phosphorylation of PLCγ1 and PKCα in addition to promoting PKCα protein stability. Moreover, we show that PLD activity is important for S6 phosphorylation and that this occurs through mTORC1. However, our data suggest that S6 phosphorylation downstream of PDGFR does not rely on Akt activation. Functionally, mTOR inhibition by rapamycin suppressed PDGF-BB-mediated cell proliferation, whereas rapamycin treatment or the loss of Rictor in the mTORC2 complex had no significant impact on the chemotactic response toward PDGF-BB.

## Results

### Inhibition of mTORC2-Akt signaling does not influence the phosphorylation of the ribosomal S6 protein downstream of mTORC1

**Figure 1:**
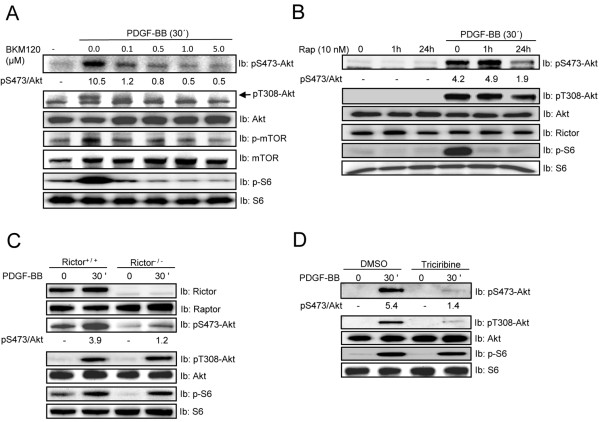
**PDGF-BB-mediated S6 phosphorylation does not require Akt phosphorylation.** NIH3T3 cells (**A, *B, D***) and Rictor-null MEFs (**C**) were serum-starved for 24 h and then stimulated for indicated time periods with PDGF-BB (20 ng/ml) without or with pretreatment for 1 hours (otherwise specified) in the presence of the inhibitors NPV-BKM120 (***A***), rapamycin (Rap, 10 nM) *(****B****)*, or tricribine (20 μM) (***D***) for indicated time periods. Total cell lysates (TCL) were prepared, and the levels of Akt phosphorylation at S473 and T308, as well as S6 phosphorylation and the expression of total protein were assayed by immunoblotting (Ib). The relative protein phosphorylations were quantified for a representative experiment.

Initially, we investigated if mTORC1 and mTORC2 function downstream of PI3K using the selective pan PI3K inhibitor NVP-BKM120, which in contrast to the classical PI3K inhibitors wortmannin or LY29004 does not inhibit mTOR [38]. NPV-BKM120 inhibited Akt phosphorylation at both Ser473 and Thr308 and also reduced mTOR and S6 phosphorylation upon PDGF-BB stimulation (Figure 1A), indicating that PI3K is required for activation of both mTOR complexes.

Previous studies have shown that Rictor is an essential component of the mTORC2 complex, which induces Akt phosphorylation at Ser473, at least in some cell types [39]. To elucidate whether mTORC2 is also necessary for PDGF-BB-induced Akt phosphorylation in fibroblasts, we used prolonged rapamycin treatment of NIH3T3 cells, which has been shown to inhibit mTORC1 and 2, as well as Rictor-deficient cells. Using both approaches, mTORC2 was found to be important for PDGF-BB-induced phosphorylation of Akt on Ser473, but not on Thr308 (Figure 1B & C), although prolonged rapamycin treatment slightly reduced Thr308 phosphorylation. In contrast, a short term treatment with rapamycin, which only inhibits mTORC1, did not influence the PDGF-BB-induced Akt phosphorylation (Figure 1B). However, the levels of Rictor were not affected by rapamycin treatment (Figure 1B).

There are reports suggesting that mTORC2-Akt can be considered as upstream regulator of mTORC1 and its downstream substrate S6 [40-42]. We investigated whether this is the case using Rictor-null cells. As can be seen in Figure 1C, no decrease in the PDGF-BB-induced S6 phosphorylation is seen in Rictor-deficient cells compared to control cells, suggesting that mTORC2-Akt is not upstream of mTORC1-S6. In contrast, both short term treatment with rapamycin (inhibits mTORC1), or long term treatment (inhibits both mTORC1 and 2) efficiently inhibited S6 phosphorylation, confirming the importance of mTORC1 for its phosphorylation (Figure 1B). To further confirm that Akt is not needed for S6 phosphorylation, we used the Akt pathway inhibitor triciribine [43]. Triciribine completely abolished the PDGF-BB-induced Akt phosphorylation, but did not influence S6 phosphorylation (Figure 1D).

To conclude, mTORC2 is of major importance for Akt Ser473 phosphorylation and the mTORC1-promoted phosphorylation of S6 is not dependent on signaling through the mTORC2-Akt pathway.

### mTORC1-mediated phosphorylation of S6 depends on PLD

**Figure 2:**
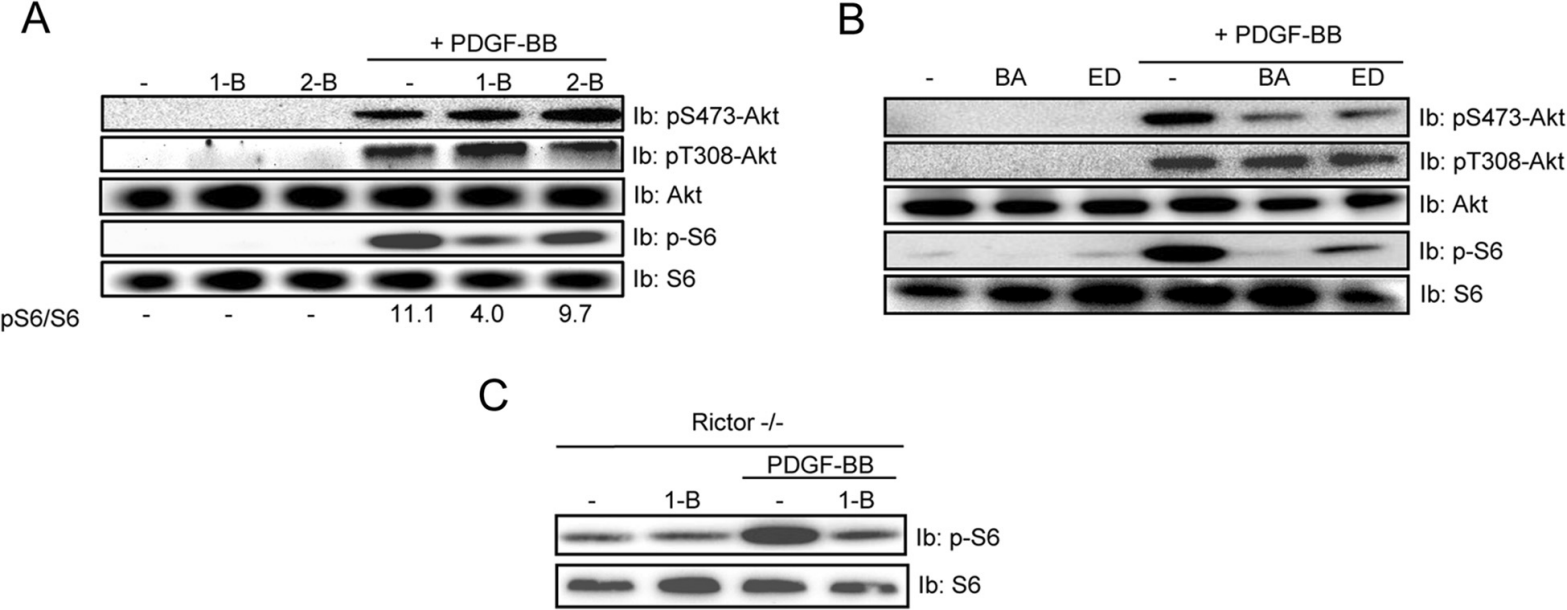
**PDGF-BB-mediated S6 phosphorylation is regulated by PLD/Ca**^**2+ **^**signaling.** NIH3T3 cells (***A, B***) and Rictor-null MEFs (***C***) were serum-starved for 24 h and then stimulated for indicated time periods with PDGF-BB (20 ng/ml) with or without pretreatment with 1-butanol (1-B, 0.3%), 2-butanol (2-B, 0.3%), and Ca^2+^ chelators BAPTA-AM (BA, 10 μM) or EDTA (ED, 2 mM) for 30 min, as indicated. Total cell lysates (TCL) were prepared, and the levels of Akt phosphorylation at S473 and T308, S6 phosphorylation, as well as the total protein expression, were assayed by immunoblotting (Ib). The relative protein phosphorylations were quantified for a representative experiment.

PLD has been proposed to contribute to mTORC1 activity by producing phosphatidic acid (PA) [35]. To investigate the importance of PLD in the activation of mTORC1 and 2, we treated cells with 1-butanol which is a preferred substrate for PLD [44], thus reducing the production of PA. The secondary alcohol, 2-butanol, was used as a negative control since PLD cannot use it as a substrate. As shown in Figure 2A, the ability of PDGF-BB to promote phosphorylation of the mTORC1 substrate S6 was reduced in the presence of 1-butanol, but not in the presence of 2-butanol. Importantly, phosphorylation of Akt, which is dependent on mTORC2, was not reduced by 1-butanol treatment (Figure 2A). Similar to NIH3T3 cells, we also found that the 1-butanol treatment attenuates S6 phosphorylation in Rictor null MEFs (Figure 1C).

Since PDGF-BB induces both Ca^2+^ influx and intracellular Ca^2+^ release [45], and it has been shown that Ca^2+^ can regulate PLD activation [46], we investigated the impact of Ca^2+^ chelators on PDGF-BB-induced S6 and Akt phosphorylation. We found that chelation of extracellular or intracellular Ca^2+^ by EDTA and BAPTA, respectively, both efficiently inhibited the phosphorylation of S6 consistent with a role for Ca^2+^ in PLD activation or subsequent mTORC1 activation (Figure 2B). Interestingly, we also observed that the PDGF-BB-induced Akt phosphorylation on Ser473 was inhibited by Ca^2+^ chelation (Figure 2B).

In summary, these finding indicate that PLD signaling is necessary for PDGF-BB-induced phosphorylation of S6 by mTORC1, and that Ca^2+^ is central for Akt phosphorylation on Ser473 in response to PDGF-BB.

### PLC signaling is important for PDGF-BB-induced Akt phosphorylation

**Figure 3:**
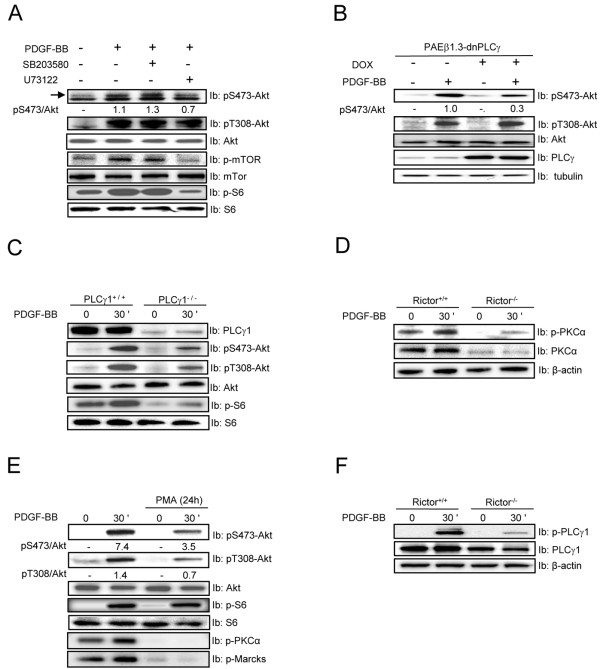
**PDGF-BB-induced Akt phosphorylation involves the PLC/PKC pathway.** NIH3T3 cells (***A*** and ***E***), dnPLCγ PAE cells (***B***), PLCγ-null (***C***) and Rictor null (***D*** and ***F***) MEFs were serum-starved for 24 h and then stimulated with PDGF-BB (20 ng/ml) with or without pretreatment with the inhibitor U73122 (5 μM) and SB203580 (10 μM) for 1 h, or with or without PMA (1 μM) for 24 h, as indicated. Akt phosphorylation level at S473 and T308, as well as mTOR, S6, PLCγ, PKCα and pMarcks phsphorylation and the total protein expression were assayed by immunoblotting (Ib) of total cell lysate. β-actin immunoblotting served as a loading control. The relative protein phosphorylations were quantified for a representative experiment.

To confirm our finding that Ca^2+^ is involved in regulation of Akt phosphorylation on Ser473, we used dominant negative PLCγ (dnPLCγ), and the low molecular weight inhibitor U73122, which inhibits both PLCγ and PLD [47,48]. Consistent with the effect of Ca^2+^ chelation (Figure 2B), U73122, as well as dnPLCγ inhibited Ser473 phosphorylation on Akt, however, no effect on the phosphorylation of Thr308 was found (Figure 3A & B). In addition, U73122 also inhibited S6 phosphorylation, in concurrence with the ability of this drug to inhibit PLD. To further investigate the role of PLCγ signaling in Akt activation, we used PLCγ1-null cells. Importantly, these cells have been shown to also have a deficient PLD activation [49]. Using these cells, we observed a defect in PDGF-BB-induced Akt phosphorylation on Ser473, but also on Thr308 (Figure 3C). This surprising finding suggests that phosphorylation of Akt on Ser473 is dependent on PLCγ activity, whereas the phosphorylation on Thr308, which is not affected by PLC inhibition or Ca^2+^ chelation, requires the presence of PLCγ1, but not necessarily its activity. Previously, it has been shown that inhibition of p38 signaling by SB203580 reduces Akt phosphorylation [50]. This effect was not observed in our experiment (Figure 3A).

Since PKC isoforms are activated downstream of PLCγ, and it has been reported that mTORC2 regulates the stability and phosphorylation of PKCα [51], we investigated if the requirement of Ca^2+^ and PLCγ for Akt phosphorylation occurred through activation of PKC. First, we confirmed the previously reported reduction of PKCα levels in the Rictor-null cells (Figure 3D). Next, we downregulated the PKC isoforms that are dependent on diacylglycerol (DAG) for their activation, by treating cells with PMA overnight. To monitor the effect of PMA treatment, we investigated phosphorylation of Myristoylated Alanine-Rich C-Kinase (MARCKS), a known PKC substrate (Figure 3E). In cells with downregulated PKC isoforms, we observed a partial reduction in the ability of PDGF-BB to promote Akt phosphorylation (Figure 3E). Consistent with our previous experiments, we found that S6 phosphorylation was independent of the reduction in Akt phosphorylation (Figure 3E).

The activity of PLCγ has been connected to its phosphorylation on Tyr783 [52]. To see if the absence of Rictor (and thus mTORC2) affects PLCγ function, we analyzed the ability of PDGF-BB to stimulate PLCγ phosphorylation. Surprisingly, we found that in Rictor-null cells the PLCγ phosphorylation was abolished and similar to what was seen for PKCα, the total protein level was slightly reduced (Figure 3F). The mechanism for the reduced PLCγ protein level is unclear, but in the case of PKCα it has been demonstrated that mTOR-mediated phosphorylation is important for protein stability [53].

To conclude, inhibition of PLCγ or Ca^2+^ chelation resulted in decreased PDGF-BB-induced phosphorylation of Akt on Ser473, but did not affect phosphorylation on Thr308. In contrast, the presence of PLCγ protein was needed for the phosphorylation on Thr308. Furthermore, we found that Rictor-null cells, which have defective PDGF-BB-induced Akt Ser473 phosphorylation, are impaired in PLCγ/PKCα signaling. However, treatment overnight with PMA inhibited Akt phosphorylation on both Ser473 and Thr308. These findings suggest that Thr308 is phosphorylated by a kinase that is downregulated by PMA treatment and thus normally regulated by DAG, possibly a novel PKC isoforms that requires DAG but not Ca^2+^. Overnight treatment with PMA did not affect PDK-1 phosphorylation and neither did PDGF-BB treatment (data not shown). In contrast, phosphorylation of Akt on Ser473 is dependent on PLCγ1 activity, Ca^2+^, DAG and the conventional PKCs.

### PDGF-BB-induced Erk1/2 MAP-kinase signaling is important for the kinetics of S6 phosphorylation

**Figure 4:**
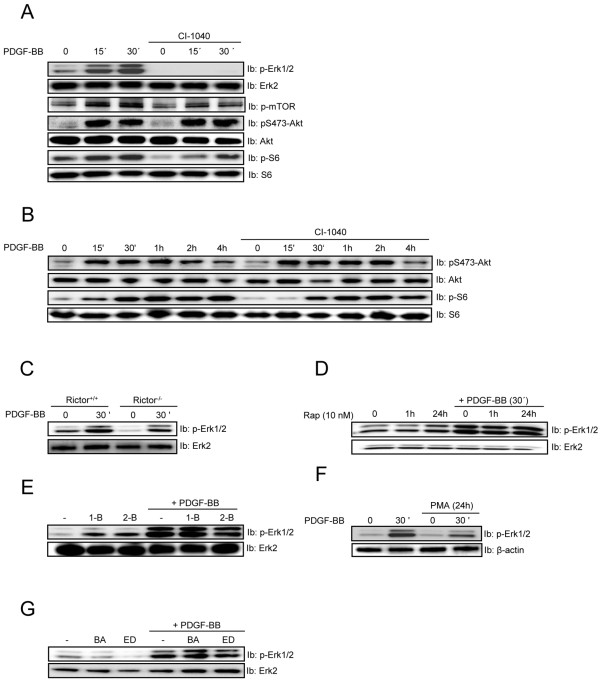
**PDGF-BB-induced Erk1/2 signalling affects the kinetics of S6 phosphorylation.** NIH3T3 cells (***A, B, D, E, *F** and ***G***) and Rictor-null MEFs (***C***) were serum-starved for 24 h and then stimulated with PDGF-BB (20 ng/ml) in the absence or presence of CI-1040 (0.5 μM) and rapamycin (10 nM) for 1 h, 1-butanol (1-B, 0.3%), 2-butanol (2-B, 0.3%), BAPTA-AM (10 μM) and EDTA (2 mM) for 30 min, and PMA (1 μM) for 24 h, as indicated. The levels of phosphorylation of Erk1/2, mTOR, Akt and S6, as well as the total protein, were assayed by immunoblotting (Ib) of total cell lysates.

In addition to Akt, MAP kinase pathways have been linked to mTOR signaling [54]. We found that the selective Mek1/2 inhibitor CI-1040 completely blocked Erk1/2 phosphorylation and reduced S6 phosphorylation, primarily after 15 min of stimulation, but had no effect on Akt phosphorylation (Figure 4A). Thus, Erk1/2 may contribute to mTORC1 activation at early stages of signaling, as previously noted [54]. To further clarify the role of Erk1/2 in mTORC1 signaling after prolonged PDGF-BB treatment, we performed a time-course experiment stimulating cells for up to 4 h (Figure 4B). We found that only the rapid, initial induction of S6 phosphorylation was inhibited by CI-1040, whereas the S6 phosphorylation reached almost the same level in cells treated with CI-1040 as in vehicle treated cells after longer time periods of PDGF-BB stimulation (Figure 4B). The PDGF-BB-induced Erk1/2 phosphorylation was not dependent on mTORC2 (Figure 4C), mTORC1 (Figure 4D & E), PKCs (Figure 4F), or the presence of Ca^2+^ (Figure 4G).

In summary, PDGF-BB-induced Erk1/2 activity is only important for the early onset of mTORC1-mediated phosphorylation of S6. Furthermore, neither mTORC1 nor mTORC2 are needed for PDGF-BB-induced Erk1/2 activation.

### Role of mTOR signaling in PDGF-BB-induced cellular responses

**Figure 5:**
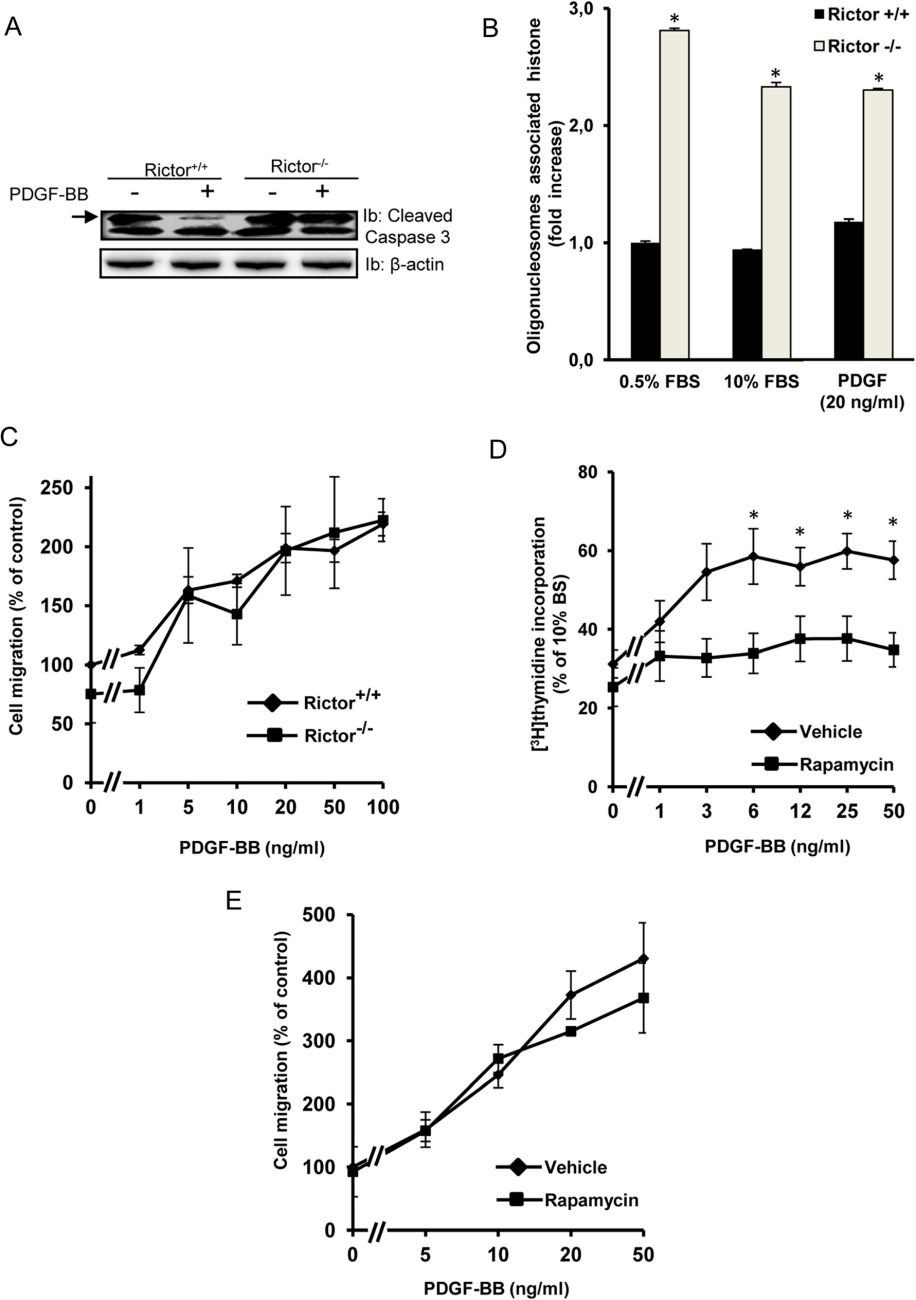
**Effect of mTOR signaling on caspase 3 cleavage, apoptosis, migration and proliferation upon PDGF-BB stimulation.** Rictor-null or control MEFs were serum-starved for 24 h and then treated with PDGF-BB for 24 h; activation of caspase 3 was measured thereafter by immunoblotting against cleaved caspase 3 (***A***). Internucleosomal DNA fragmentation was quantitatively determined by assaying for cytoplasmic mononucleosome- and oligonucleosome-associated histone accumulated in apoptotic cell (***B***), data represent three separate experiments each performed in duplicate ± SEM. Cell migration experiments were carried out in a 96-well ChemoTX cell migration microplate. The wells of the microplate were filled with medium containing combinations of PDGF-BB with Rictor-null or control MEFs (***C***), as well as NIH3T3 cells with or without longterm treatment with rapamycin (***E***), as indicated. The amounts of migrated cells are given as index units; data represent three separate experiments, each performed in quadruplicates ± SEM. In separate experiments, NIH3T3 cells were serum-starved and then stimulated for 24 h with PDGF-BB in medium containing [^3^H] thymidine. The fold increase of PDGF-induced [^3^H]thymidine incorporation over the respective positive control values is shown. Values are means ± S.E of three independent experiments each performed in triplicate. Statistical significant differences (Students *T*-test) are indicated by *P < .05 compared with unstimulated or control cells (***B*** &***D***).

Next, we wanted to elucidate the functional consequences of interfering with mTOR signaling for PDGF-BB-mediated cellular responses, i.e. survival, migration and proliferation. To this end, we used the Rictor-null cells which lack a functional mTORC2 complex, as well as long term treatment (24 h) with rapamycin to inhibit both mTORC1 and 2. We found that serum starvation induced caspase-3 cleavage, which could be rescued by addition of PDGF-BB in control cells, but not in Rictor null cells, suggesting a role of mTORC2 in promoting cell survival in response to PDGF-BB (Figure 5A). In accordance with a recent report [55] we could confirm that Rictor-null cells have increased rate of apoptosis compared to control MEFs (Figure 5B). Interestingly, in these cells the apoptosis could not be modulated by either serum levels or addition of PDGF, despite the reduction of caspase 3 cleavage observed in control MEFs in the presence of PDGF. The reasons of these findings remain to be elucidated.

In contrast, the migratory response was not affected by loss of the mTORC2 complex (Figure 5C). As expected, downregulation of both mTORC1 and 2 by rapamycin strongly inhibited PDGF-BB-promoted DNA synthesis in NIH3T3 cells (Figure 5D). Unfortunately, we were not able to analyze the proliferation of Rictor-null cells in response to PDGF-BB, since neither control nor knock-out cells responded to PDGF-BB in the proliferation assay (data not shown). Furthermore, long term treatment with rapamycin did not affect the PDGF-BB-induced migration of NIH3T3 cells (Figure 5E).

In conclusion, PDGF-BB signaling through mTORC2 is important for the ability of PDGF-BB to suppress starvation-induced cleavage of caspase 3, but not for chemotaxis. Complete inhibition of mTOR signaling by rapamycin abolished the ability of PDGF-BB to promote cell proliferation.

## Discussion

Akt is an important kinase mediating survival signaling, which is regulated by phosphorylation on Thr308 by PDK1 and on Ser473 by several other kinases. A large number of kinases have been proposed to perform the Ser473 phosphorylation [56]. In the present study, we showed that phosphorylation of Akt on Ser473 in response to PDGF-BB was critically dependent on the mTORC2 complex since the phosphorylation was strongly repressed in Rictor-null cells. Consistently, prolonged treatment with rapamycin that downregulates both mTORC1 and 2, inhibited the PDGF-BB-induced phosphorylation on Ser473, whereas short term rapamycin treatment which only inhibits mTORC1, did not. Furthermore, we also found that U73122, which blocks both PLC and PLD activities, as well as Ca^2+^ chelating agents, inhibited the PDGF-BB-mediated phosphorylation of Akt on Ser473, but not on Thr308. It has been reported, and we confirmed, that in Rictor-null cells the level of PKCα is severely reduced [51]. In addition, we found that PLCγ phosphorylation is dramatically suppressed in Rictor null cells compared to control cells. Interestingly, treatment with PMA overnight to downregulate DAG-dependent PKC isoforms resulted in inhibition of phosphorylation of Akt on both Ser473 and Thr308. The effect on Thr308 did not occur by any reduction in p-PDK1 levels, indicating that a DAG responsive kinase is involved in the phosphorylation of Thr308. Another possibility is that while PMA treatment overnight did not affect the phosphorylation of PDK1, it may have influenced its intracellular localization. We also found that in PLCγ1 null cells, the phosphorylation of both Ser473 and Thr308 on Akt were reduced. Interestingly, it has recently been demonstrated that PDK1 and PLCγ interact after EGF stimulation and that PDK1 is involved in the activation of PLCγ in a manner that only partially depends on PDK1 activity [57]. Thus, it is possible that the interaction between PDK1 and PLCγ regulates the ability of PDK1 to phosphorylate Akt on Thr308, potentially by acting as a scaffold. This hypothesis is consistent with our observation that PDGF-BB-induced Thr308 phosphorylation is reduced in PLCγ deficient cells but is not affected by PLCγ inhibition or Ca^2+^ chelation.

Collectively, these results suggest that the pathway leading from the PDGFR to phosphorylation of Akt involves mTORC2 and PLC/Ca^2+^ signaling, although some aspects of the molecular mechanism remain to be elucidated. Activation of Akt has been associated with increased cell viability [58]. Consistent with a critical role for mTORC2 in Akt activation, we found that in Rictor-deficient cells, which are blunted in their ability to activate Akt, PDGF-BB was not able to suppress starvation-induced caspase-3 cleavage, whereas it did so in control cells.

mTORC1 is widely accepted to be responsible for S6-kinase activation leading to phosphorylation of the ribosomal S6 protein, thus facilitating protein translation. Several reports have suggested that mTORC1 may be downstream of Akt signaling [13], although this has been challenged [59]. Our results suggest that in PDGF-BB-stimulated fibroblasts, Akt is not upstream of S6 phosphorylation; for example, in Rictor-null cells, where Akt phosphorylation on Ser473 is reduced, S6 phosphorylation was normal. Moreover, treating cells with the Akt pathway inhibitor triciribine completely abolished Akt phosphorylation, but had no impact on PDGF-BB promoted S6 phosphorylation. This is consistent with a study in Drosophila showing that Akt phosphorylation of TSC2 is not required for mTOR activation [60], but in contrast to studies on insulin signaling, where it was shown that Akt phosphorylation of TSC2 is necessary for mTORC1 activation [9].

We observed inhibition of S6 phosphorylation after treatment with Ca^2+^ chelators. A possible Ca^2+^-dependent pathway from the PDGFR to mTORC1 involves PLD. PLD degrades phosphatidylcholine into choline and phosphatidic acid. Phosphatidic acid have been shown to bind to mTOR and activate mTORC1 [35]. Treatment of cells with the PLD inhibitor 1-butanol suppressed the PDGF-BB-induced S6 phosphorylation, without affecting Akt phosphorylation. As expected, the PLC/PLD inhibitor U73122 also suppressed S6 phosphorylation. It is possible that PLCγ contributes to PLD activation by causing increased Ca^2+^ levels, since PLD requires Ca^2+^ for its activity [61]. In support of this notion, it has been reported that in PLCγ-deficient cells, PLD signaling is reduced and this may explain the observed reduction in S6 phosphorylation in PLCγ1^−/−^ cells. Analogous to Akt activation where both mTORC2 and PDK1 phosphorylation are required for full Akt activation, mTORC1 has been proposed to collaborate with PDK1 in S6 kinase activation [62].

Erk1/2 MAP-kinases are activated by most receptor tyrosine kinases and have been shown to regulate proliferation as well as protein translation [63]. mTOR is also involved in these processes, and there are reports implicating a link between Erk1/2 and mTOR signaling. In particular, it has been shown that Erk1/2 can directly phosphorylate Raptor and as a consequence activate mTORC1 [64]. In addition, both Erk1/2 and the downstream p90 ribosomal S6 kinase can phosphorylate the TSC1/2 complex resulting in mTORC1 activation [65]. To explore whether Erk1/2 is involved in PDGF-BB-induced mTOR signaling, we investigated the effect of the selective MEK1/2 inhibitor CI-1040 on Akt and S6 phosphorylation. Inhibition of the Erk1/2 pathway did not influence the PDGF-BB-induced phosphorylation of Akt, however, it delayed the onset of S6 phosphorylation. Conversely, interfering with mTOR signaling did not significantly affect the PDGF-BB-induced Erk1/2 phosphorylation. Thus, signaling through the Erk1/2 pathway is not critical for mTORC2 activity, but is required for the initial rapid onset of mTORC1. The S6 phosphorylation observed after prolonged PDGF-BB treatment was not dependent on Erk1/2 signaling. Furthermore, it has been proposed that inhibition of mTOR-dependent signaling by rapamycin leads to an increased Erk1/2 activity and potentiation of PDGF-induced Erk1/2 phosphorylation [66]. In contrast to these findings, we observed that neither interfering with mTOR signaling using Rictor-null cells, short or long term treatment of NIH3T3 cells with rapamycin and PLD inhibition, nor Ca^2+^ chelation affected PDGF-BB-induced Erk1/2 phosphorylation.

Signaling through mTOR has been reported to regulate both proliferation and migration [13,67]. A commonly used inhibitor of mTOR is rapamycin. However, the two mTOR containing complexes, mTORC1 and mTORC2, have different sensitivities to rapamycin. mTORC1 is rapidly inhibited whereas mTORC2 requires prolonged rapamycin treatment; thus, short term (1 h) treatment with rapamycin only inhibits mTORC1 whereas long term (24 h) treatment also inhibit mTORC2. Treating cells for extended time periods with rapamycin abolished the mitogenic effect of PDGF-BB, suggesting that functional mTOR signaling is required for cell proliferation. In contrast, Rictor-deficient cells showed a similar chemotactic response as control cells towards PDGF-BB, indicating that mTORC2 is not involved in PDGF-BB-dependent cell migration; this is surprising since mTORC2 has been shown to regulate cell polarity and the dynamics of the actin cytoskeleton [68], although no alterations in the actin cytoskeleton were observed in Rictor-null MEFs [51,59]. Similarly, inhibition of mTORC1 and 2 in NIH3T3 cells did not influence the chemotactic properties of these cells. mTORC2 may affect cell migration by promoting PKCα-dependent phosphorylation of the focal adhesion component paxillin [20]. However, it has previously been found that PDGF-BB can promote paxillin phosphorylation through the JNK MAP kinase pathway [69], and this may relieve the absolute requirement of mTORC2 in PDGF-BB-mediated fibroblast migration.

## Conclusions

**Figure 6:**
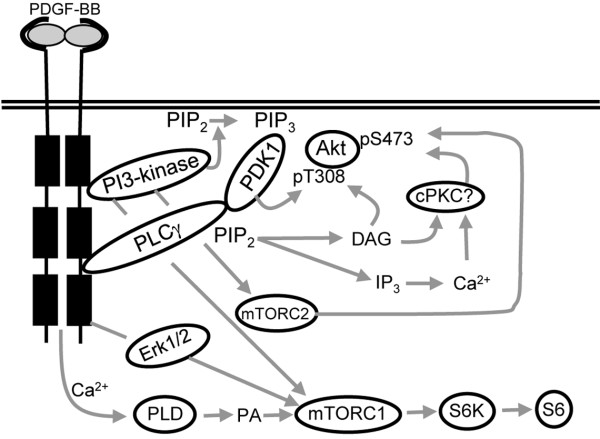
**Schematic representation of PDGF-BB-mediated regulation of Akt and S6.** Initially, PDGF-BB-mediated activation of Akt involves both mTORC2 and the PLCγ/PKC pathways. Activation of S6 downstream of mTORC1 depends on PLD activation, independent of mTORC2 and Akt signaling. Ca^2+^ is required for regulation of Akt and S6 activity.

The pathway from PDGFR leading to phosphorylation of Akt involves both the mTORC2 and PLCγ/PKC pathways. In contrast, phosphorylation of S6 downstream of mTORC1 depends on PLD activation, but is independent of mTORC2 and Akt signaling (Figure 6). During conditions where Erk1/2 signaling is inhibited, the initial S6 phosphorylation is delayed. Interfering with mTOR signaling did not affect PDGF-BB-induced Erk1/2 phosphorylation. Functionally, inhibition of mTORC1 and 2 by rapamycin effectively blocked PDGF-BB-mediated cell proliferation. Figure 6 depicts a schematic figure of key roles of mTOR in PDGF-BB-induced cell signaling.

## Materials and methods

### Reagents

Recombinant human PDGF-BB was generously provided by Amgen (Thousand Oaks, CA). The inhibitors CI-1040 (PD184352), triciribine and NVP-BKM120 were from Calbiochem (San Diego, CA), Cayman Chemical Company (Michigan, USA) and Selleckchem (Houston, USA), respectively. Antibodies against phosphorylated Akt (#9271), phosphorylated mTOR (#5536), phosphorylated S6 (#4858), cleaved caspase 3 (#9661), phosphorylated Erk1/2 (#9106) and phospho-MARCKS (#2741) were purchased from Cell Signaling Technology (Beverly, MA). A β-actin antibody was purchased from Sigma (St. Louis, MI). A rabbit antiserum recognizing Erk was raised against a peptide corresponding to the carboxyl-terminal sequence EETARFQPGYRS conjugated to KLH.

The wild-type control and Rictor-knockout mouse embryonic fibroblasts (MEFs) have been described previously [59] and were kindly provided by Dr Mark Magnuson (Vanderbilt University School of Medicine, Nashville, TN, U.S.A.). PLCγ1-null MEFs have been described previously [70] and were kindly provided by Dr Matilda Katan (Institute for Cancer Research, London, UK).

### Cell culture

The murine embryonic fibroblast cell line NIH3T3, and MEFs were cultured in Dulbecco's modified Eagle's medium (DMEM) with 10% bovine serum, 100 U/ml penicillin and 100 μg/ml streptomycin. For serum-starvation, cells were washed once and incubated in medium containing 0.1% FBS. Lipase inactive PLC-γ1 H335F/H380F (dnPLCγ), porcine aortic endothelial (PAE) cells were cultured in Ham’s F-12 containing 10% bovine serum albumin, in the presence or absence of 20 ng/ml doxycycline to induce protein expression [71].

### Immunoblotting

Subconfluent cells were starved and incubated with vehicle or inhibitors at the indicated concentrations and thereafter stimulated with PDGF-BB (20 ng/ml, or as specified) for the indicated periods of time. Cells were washed two times in ice-cold phosphate-buffered saline and lysed in 20 mM Tris pH 7.4, 150 mM NaCl, 5 mM EDTA, 1% Triton X-100, 0.1% SDS, 1% deoxycholate, 1 mM Pefa Bloc and 1 mM sodium orthovanadate. Extracts were clarified by centrifugation, and protein concentration was determined by the BCA protein assay (Pierce). Equal amounts of lysates were boiled with SDS sample buffer containing dithiothreitol. Proteins were separated by SDS-PAGE and then electro-transferred to polyvinylidene difluoride membranes (Immobilon P), which were blocked in 5% bovine serum albumin or 5% milk in Tris-buffered saline solution containing 0.1% Tween-20. Primary antibodies were diluted according to the manufacturer’s instructions and membranes incubated overnight at 4°C. After washing, the membranes were incubated with horseradish peroxidase-conjugated anti-rabbit or anti-mouse IgG antibodies (both from Amersham Biosciences), and proteins were visualized using ECL immunoblotting detection systems from Roche Applied Science on a cooled charge-coupled device (CCD) camera (Fuji, Minami-Ashigata, Japan). Densitometrical analysis of the immunoblots was performed using advanced image data analyzer (AIDA) software (Fujifilm).

### Apoptosis assay

Control and Rictor-null MEFs were starved for 24 h, then the extent of apoptosis was determined by quantification of nucleosomes released into the cytoplasma using the Cell Death Detection ELISA Plus kit (Roche Applied Science) according to the manufacturer's directions. In the separate experiments the level of caspase-3-cleaved fragments was analyzed by immunoblotting.

### [^3^H]thymidine incorporation assay

For thymidine incorporation assay subconfluent cell cultures were serum-starved in 24-well plates and then incubated for 24 h in the presence or absence of rapamycin with PDGF-BB in DMEM containing [^3^H]thymidine (0.1 μCi/ml). Incorporation of ^3^H-radioactivity into acid-insoluble material was measured by a scintillation counter. The obtained count per minute values in triplicate was normalized against the positive control of cultures incubated in 10% bovine serum for each experiment.

### Cell migration assays

Cell migration was determined as previously described [69]. In brief, 96-well ChemoTX (Neuroprobe, Gaithersburg, MD) cell migration microplate filters were coated with 50 μg/ml fibronectin (BD Biochemicals, Erembodegem, Belgium) for 1 h at room temperature. Control and Rictor null-MEFs, or NIH3T3 cells treated with or without rapamycin, were serum-starved overnight and then trypsinized into single cells. The wells of the ChemoTX microplate were filled with DMEM containing the indicated PDGF-BB concentrations. The filters were placed in the wells and 50,000 cells were added on top of each filter. The chamber was incubated for 4 h at 37°C, 5% CO_2_. Cells adhering to the bottom of the filter were fixed by a 3-min incubation in 96% ethanol. The adherent cells were stained with Giemsa (Sigma)-and the migration indices were assessed by scanning the filter in a CCD camera (Fuji). Quantifications were performed using Aida Image Analyzer software. All experiments were performed in quadruplicate, and single representative data of three separate experiments ± SD are shown.

## Abbreviations

Erk: Extracellular regulated kinase; MAPK: Mitogen activated protein kinase; MEF: Mouse embryo fibroblast; mTOR: Mammalian target of rapamycin; PDGF: Platelet-derived growth factor; PDGFR: PDGF receptor; PLCγ1: Phospholipase C gamma 1; PI3K: Phosphatidylinositol 3-kinase.

## Competing interests

The authors declare that they have no competing interests.

## Authors’ contributions

MR, planned and performed experiments, analyzed the data and contributed to manuscript preparation; CHH, planned experiments, analyzed data and contributed to manuscript preparation; JL, planned and performed experiments, analyzed the data and contributed to manuscript preparation. All authors read and approved the final manuscript.
